# County Provision for the Insane

**Published:** 1885-11

**Authors:** 


### EDITORIAL.

---

COUNTY PROVISION FOR THE INSANE.

DR. JAMES G. KIERNAN read a paper, entitled COUNTY PROVISION FOR THE INSANE, before the CHICAGO MEDICAL SOCIETY, on Monday evening, nineteenth October, 1885. The paper appears in full in this issue of the JOURNAL.

We desire to call attention to DR. KIERNAN'S timely suggestions. The discussion of the subject is impersonal and fair. On motion, a committee was appointed by the PRESIDENT of the CHICAGO MEDICAL SOCIETY to investigate the condition of the COOK COUNTY INSANE ASYLUM.

On Saturday night, twenty-fourth October, a meeting of citizens to discuss the present condition and alleged mismanagement of the COOK COUNTY INSANE ASYLUM was held at the Sherman House. The meeting was a representative one.

A committee was appointed to investigate the alleged mismanagement, collect evidence and report at a public meeting.

The profession and public generally expect positive results from the investigations of these two committees. Up to the present time, all attempts at inquiry have proved abortive. The causes of failure are not obscure. Inattention to duty may be mentioned as an important one. The methods of investigation, practiced under such conditions in English insane asylums, are worthy of serious consideration.

